# Coronary artery restenosis and target lesion revascularisation in women by pregnancy history

**DOI:** 10.1136/openhrt-2022-002130

**Published:** 2023-03-17

**Authors:** Moa Pehrson, Andreas Edsfeldt, Giovanna Sarno, Abigail Fraser, Janet W. Rich-Edwards, Isabel Goncalves, Mats Pihlsgård, Simon Timpka

**Affiliations:** 1 Perinatal and Cardiovascular Epidemiology, Lund University Diabetes Centre, Department of Clinical Sciences Malmö, Lund University, Malmö, Sweden; 2 Cardiovascular Research- Translational Studies, Lund University, Malmö, Sweden; 3 Wallenberg Center for Molecular Medicine, Lund University, Lund, Sweden; 4 Department of Cardiology, Skåne University Hospital, Lund/Malmö, Sweden; 5 Department of Medical Sciences, Cardiology and Uppsala Clinical Research Center, Uppsala University, Uppsala, Sweden; 6 Population Health Science, Bristol Medical School, University of Bristol, Bristol, UK; 7 Division of Women's Health, Department of Medicine, Brigham and Women’s Hospital and Harvard Medical School, Boston, Massachusetts, USA; 8 Department of Obstetrics and Gynaecology, Skåne University Hospital, Malmö, Sweden

**Keywords:** coronary artery disease, percutaneous coronary intervention, epidemiology, pregnancy

## Abstract

**Background:**

Women’s pregnancy history is associated with incident risk of coronary artery disease with some evidence also suggesting a relevance for prognosis following treatment.

**Objectives:**

To study the associations between maternal history of preterm delivery, a history of small for gestational age infant, parity and age at first delivery with clinical restenosis after percutaneous coronary intervention (PCI).

**Methods:**

In this prospective cohort study, we included 6027 women <65 years undergoing their first PCI 2006–2017, merging clinical register data on PCI procedures in Sweden with comprehensive registry data on deliveries since 1973. We used proportional hazards regression to study the association between aspects of pregnancy history and clinical restenosis in per-segment analyses, and with target lesion revascularisation (TLR) in per-patient analyses. We adjusted models for procedural-related and patient-related predictors of restenosis.

**Results:**

During 15 981 segment-years of follow-up, 343 (3.7%) events of clinical restenosis occurred. We found no strong evidence of associations between the studied aspects of pregnancy history and clinical restenosis following PCI. For example, the restenosis HR for a history of preterm delivery in the fully adjusted model was 1.09 (95% CI 0.77 to 1.55) and the TLR HR was 1.18 (95% CI 0.91 to 1.52).

**Conclusion:**

Risk of restenosis following treatment with PCI did not differ by the studied aspects of pregnancy history, including preterm delivery, in young and middle-aged women. Larger studies are needed to obtain more precise estimates.

**WHAT IS ALREADY KNOWN ON THIS TOPIC**

- Pregnancy history, including adverse pregnancy outcomes, is associated with future coronary artery disease risk. A history of preterm delivery is associated with an increased risk of adverse outcomes following coronary artery stenting.

**WHAT THIS STUDY ADDS**

- We found no strong evidence that the studied aspects of pregnancy history, including preterm delivery, were associated with risk of restenosis following treatment with percutaneous coronary intervention in young and middle-aged women.

**HOW THIS STUDY MIGHT AFFECT RESEARCH, PRACTICE OR POLICY**

- Based on our results, pregnancy history should not be clinically considered in the management of women requiring percutaneous coronary intervention. Larger studies are needed to obtain more precise estimates.

## Introduction

Female sex is associated with worse outcome following percutaneous coronary intervention (PCI).^1^ A woman’s pregnancy history, including pregnancy complications such as preterm delivery (PTD), small for gestational age infant (SGA), age at first delivery and total parity, is associated with her future risk of coronary artery disease (CAD).^2–4^ However, there are indications that the pregnancy history also is associated with women’s prognosis following treatment of CAD with PCI. We have previously shown that women with a history of PTD have an increased risk of major adverse cardiovascular events following coronary artery stenting compared with women who delivered at term.^5^ Studying restenosis risk among women with a history of hypertensive disorders of pregnancy, we found that late-onset pre-eclampsia is associated with a reduced risk following PCI.^6^ While restenosis remains a common complication of PCI,^7^ it is not known the extent to which other aspects of pregnancy history are associated with restenosis risk.

Here, we aimed to study association between aspects of pregnancy history and risk of symptomatic (ie, clinical) restenosis following PCI. In order to do so, we merged clinical register data on women undergoing PCI procedures in Sweden with comprehensive data on PTD history, SGA, age at first delivery and total parity. As each woman can have had more than one coronary artery segment targeted with PCI during the same procedure, we complementary studied the risk of clinical restenosis per segment and the risk of target lesion revascularisation (TLR) per patient.

## Methods

**Figure 1:**
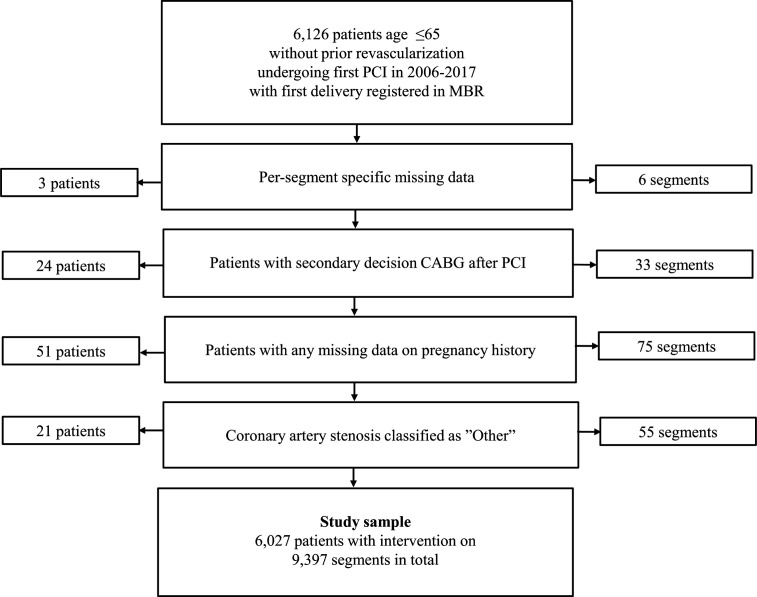
Flow chart of study sample. Figure shows the inclusion and exclusion criteria for the study sample. CABG, coronary artery bypass; PCI, MBR, Medical Birth Register; PCI, percutaneous coronary intervention.

We conducted a prospective cohort study on a national sample originating from two comprehensive Swedish registers: Swedish Coronary Angiography and Angioplasty Registry (SCAAR) and the Medical Birth Register from the National Board of Health and Welfare. The cohort and statistical methodology have previously been described.^6^ In short, women were included based on the following criteria: PCI procedure recorded in SCAAR in 2006–2017 after first pregnancy and first pregnancy recorded in the Medical Birth Register (figure 1). We excluded procedures before 2006 as not all covariables were routinely registered before that time point. As age is an important factor to consider for the prognosis following PCI and the inclusion of older women was limited by the lack of delivery data prior to the start of registry registration in 1973, we excluded women >65 years at time of index PCI. We also excluded individuals with missing data on any segment variable (n=3), individuals with a history of coronary artery bypass (CABG) or a planned CABG procedure at time of index PCI (n=24), individuals with missing data on any exposure variables (n=51) and/or individuals with coronary artery stenosis classified as ‘other’ (n=21).

Data on migration and death during follow-up originated from Statistics Sweden and the Swedish National Death Register, respectively. The information was linked using the Swedish identification number.^8^

### Pregnancy history

Data on pregnancy history originated from the Swedish Medical Birth Register. The Medical Birth Register has collected data on most pregnancies in Sweden since 1973.^9^ PTD was defined as delivery <36+6 weeks of gestation and further subcategorised into late PTD (34+0–36+6 weeks of gestation) and early PTD (22+0–33+6 weeks of gestation). PTD history was further defined according to the woman’s most PTD prior to her first PCI procedure. Parity was categorised as 1, 2–3 and >4, and based on all pregnancies before index PCI procedure.^3^ Any SGA was defined as >2 SDs below the normal weight by infant sex and length of pregnancy.^10^ Age at first delivery was categorised as <20 years, 20–34 years and >35 years.^2^

### Index PCI procedure

Data on the index PCI procedure originated from SCAAR, a national quality of care register aiming to record information on all coronary angiographies and PCI procedures in Sweden.^11 12^ Each procedure in SCAAR is described with angiographic, demographic and procedure-related variables. We included several variables as procedural predictors of restenosis. Indication for PCI was categorised as ST-elevation myocardial infarction, non-ST-elevation myocardial infarction, unstable CAD, stable CAD and other. We grouped coronary segments identified in SCAAR into specific vessels treated: left main stem, left anterior descending artery, right coronary artery, left circumflex coronary artery and other. Type of device(s) used were grouped into the following categories: bare metal stent, bare metal stent with balloon predilation, drug-eluting stent, drug-eluting stent with balloon predilation, drug coated balloon and not drug coated balloon. We also included length of stent and stent diameter >3 mm following procedure, using interaction terms with stent as the variables are only applicable to these procedures. In addition to these procedure-related variables, we included patient-related predictors for restenosis. Hypertension and/or dyslipidaemia was defined as a woman receiving antihypertensive treatment or lipid-lowering agents at time of PCI, respectively. Diabetes was defined as a patient having a known diabetes diagnosis at time of PCI, regardless of treatment. Smoking status was divided into the categories never smoker, ex-smoker or current smoker at time of PCI. Prior myocardial infarction was defined by SCAAR as myocardial infarction prior to the current hospitalisation, including silent myocardial infarction based on electrocardiography or echocardiography findings. To account for general improvement of care during the study period, we also included year of procedure and categorised it into three categories: 2006–2009, 2010–2013 and 2014–2017.

### Clinical endpoints

Restenosis was defined as in the SCAAR registry: a stenosis assessed by angiographic visual estimation (>50%) or by fractional flow reserve <0.80 in a previously stented segment identified by coronary angiography for any clinical indication performed anywhere in Sweden.^13 14^

TLR was defined as repeated PCI targeting any segment included in the index procedure or CABG after the index procedure, whichever came first. Information on repeat PCI was obtained from SCAAR and CABG procedures from the Swedish in-patient care registry. To minimise the risk of including planned CABG procedures as events individuals with a planned CABG procedure at time of index PCI were excluded as described above.

### Statistical analysis

We summarised characteristics of the study sample as means or percentages and calculated the percentages of missing data for each variable. Event rates were estimated using the Kaplan-Meier method and comparisons made using the log-rank test.

To study pregnancy history as a prognostic maker of clinical restenosis we used proportional hazards regression in a per-segment analysis. To account for dependence between coronary artery segments in the same patient, we used a Jackknife (on patient level) estimator of variance. We adjusted for risk factors of restenosis in three steps. In model I, we included age at index PCI, in model II, we additionally accounted for procedure related variables, and in model III, we additionally adjusted for patient-related variables. Right-censoring during follow-up occurred at 2 years of follow-up, end of follow-up in 2017, migration out of Sweden or death, whichever came first.

To study pregnancy history as a prognostic marker of TLR, we used proportional hazards regression in a per-patient analysis. We adjusted for prognostic factors as described above for clinical restenosis with some exceptions. We did not include segment specific variables such as type of device used and class of stenosis. Furthermore, each treated vessel category was included as a not mutually exclusive binary variable, and we additionally adjusted for number of treated vessels. We repeated the analysis for each pregnancy history exposure variable. Right-censoring during follow-up occurred at 2 years of follow-up, end of follow-up in 2017, migration out of Sweden or death, whichever came first.

Multiple imputation was used to impute missing data on a patient level. Individuals with any missing data on a segment level were excluded as described above and 20 imputed datasets were created using multiple imputation by chained equations. Results were combined using PROC MIANALYZE. Statistical analyses were conducted using SAS V.9.4. A significance level of p<0.05 was used for hypothesis testing.

## Results

Our study sample consisted of 6027 women with 9397 segments (figure 1). Median time from first delivery to index procedure was 31.0 years (IQR 25.0–35.4 years). Table 1 shows patient characteristics of the study sample by pregnancy history, and online supplemental table 1 shows segment characteristics of the study sample by pregnancy history. In short, women with a history of PTD more often presented with diabetes, hypertension and hyperlipidaemia at the time of PCI compared with women without a history of PTD. Women with a history of SGA were more often active smokers and presented with ST-elevation myocardial infarction (STEMI) at the time of PCI compared with women with no history of SGA. Women unipara at the time of PCI more often presented with diabetes compared to multiparous women. Women who experienced their first delivery at a younger age more often presented with STEMI at the time of PCI compared with women who experienced their first delivery at age >35 years. Segment characteristics were similar for all exposures.

10.1136/openhrt-2022-002130.supp1 Supplementary data

**Table 1:** Patient characteristics per patient at time of index PCI by aspects of pregnancy history (n=6027)

	Preterm delivery		Small for gestational age		Parity at time of PCI			Age at first delivery (years)			
Variables n, (%) unless stated	No PTD (n=5022)	Ever PTD (n=1005)	No SGA (n=5324)	Ever SGA (n=703)	Parity 1 (n=1258)	Parity 2–3 (n=4165)	Parity >4 (n=604)	Age <20 (n=788)	Age 20–34 (n=4954)	Age >35 (n=285)	
											**Missing (%)**
Age (SD)	55.3 (6.4)	54.0 (6.8)	55.1 (6.5)	54.9 (6.5)	55.8 (6.7)	55.0 (6.5)	54.3 (5.9)	53.4 (5.7)	55.3 (6.5)	55.4 (6.7)	–
Previous MI	177 (3.5)	55 (5.5)	191 (3.6)	41 (5.8)	63 (5.0)	154 (3.7)	15 (2.5)	37 (4.7)	182 (3.7)	13 (4.6)	1.3
Diabetes	707 (14.1)	237 (23.6)	852 (16.0)	92 (13.1)	269 (21.4)	577 (13.9)	98 (16.2)	138 (17.5)	761 (15.4)	45 (15.8)	0.5
Hypertension	2179 (43.4)	479 (47.7)	2350 (44.1)	308 (43.8)	584 (46.4)	1823 (43.8)	251 (41.6)	356 (45.2)	2180 (44.0)	122 (42.8)	1.5
Dyslipidaemia	1457 (29.0)	356 (35.4)	1616 (30.4)	197 (28.0)	425 (33.8)	1202 (28.9)	186 (30.8)	250 (31.7)	1474 (29.8)	89 (31.2)	1.8
Smoking											3.5
Never	1422 (28.3)	276 (27.5)	1544 (29.0)	154 (21.9)	337 (26.8)	1228 (29.5)	133 (22.0)	98 (12.4)	1489 (30.1)	111 (39.0)	
Ex-smoker	1206 (24.0)	223 (22.2)	1283 (24.1)	146 (20.8)	309 (24.6)	999 (24.0)	121 (20.0)	159 (20.2)	1205 (24.3)	65 (22.8)	
Smoker	2218 (44.2)	469 (46.7)	2308 (43.4)	379 (53.9)	554 (44.0)	1801 (43.2)	332 (55.0)	507 (64.3)	2083 (42.1)	97 (34.0)	
Year of index PCI											–
2006–2009	1315 (26.2)	239 (23.8)	1355 (25.5)	199 (28.3)	356 (28.3)	1066 (25.6)	132 (21.9)	184 (23.4)	1278 (25.8)	92 (32.3)	
2010–2013	1685 (33.6)	351 (34.9)	1792 (33.7)	244 (34.7)	411 (32.7)	1413 (33.9)	212 (35.1)	257 (32.6)	1700 (34.3)	79 (27.7)	
2014–2017	2022 (40.3)	415 (41.3)	2177 (40.9)	260 (37.0)	491 (39.0)	1686 (40.5)	260 (43.1)	347 (44.0)	1976 (39.9)	114 (40.0)	
Indication for PCI											–
STEMI	1843 (36.7)	359 (35.7)	1902 (35.7)	300 (42.7)	446 (35.5)	1540 (37.0)	216 (35.8)	297 (37.7)	1810 (36.5)	95 (33.3)	
NSTEMI	696 (13.9)	131 (13.0)	732 (13.8)	95 (13.5)	161 (12.8)	565 (13.6)	101 (16.7)	136 (17.3)	653 (13.2)	38 (13.3)	
Unstable CAD	1614 (32.1)	326 (32.4)	1735 (32.6)	205 (29.2)	421 (33.5)	1316 (31.6)	203 (33.6)	228 (28.9)	1613 (32.6)	99 (34.7)	
Stable CAD	715 (14.2)	150 (14.9)	782 (14.7)	83 (11.8)	189 (15.0)	606 (14.6)	70 (11.6)	98 (12.4)	722 (14.6)	45 (15.8)	
Other	154 (3.1)	39 (3.9)	173 (3.3)	20 (2.8)	41 (3.3)	138 (3.3)	14 (2.3)	29 (3.7)	156 (3.2)	8 (2.8)	

Information presented per patient.

CAD, coronary artery disease; MI, myocardial infarction; NSTEMI, non-ST-elevation myocardial infarction; PCI, percutaneous coronary intervention; PTD, preterm delivery; SGA, small for gestational age; STEMI, ST-elevation myocardial infarction.

### Clinical restenosis by pregnancy history

**Figure 2:**
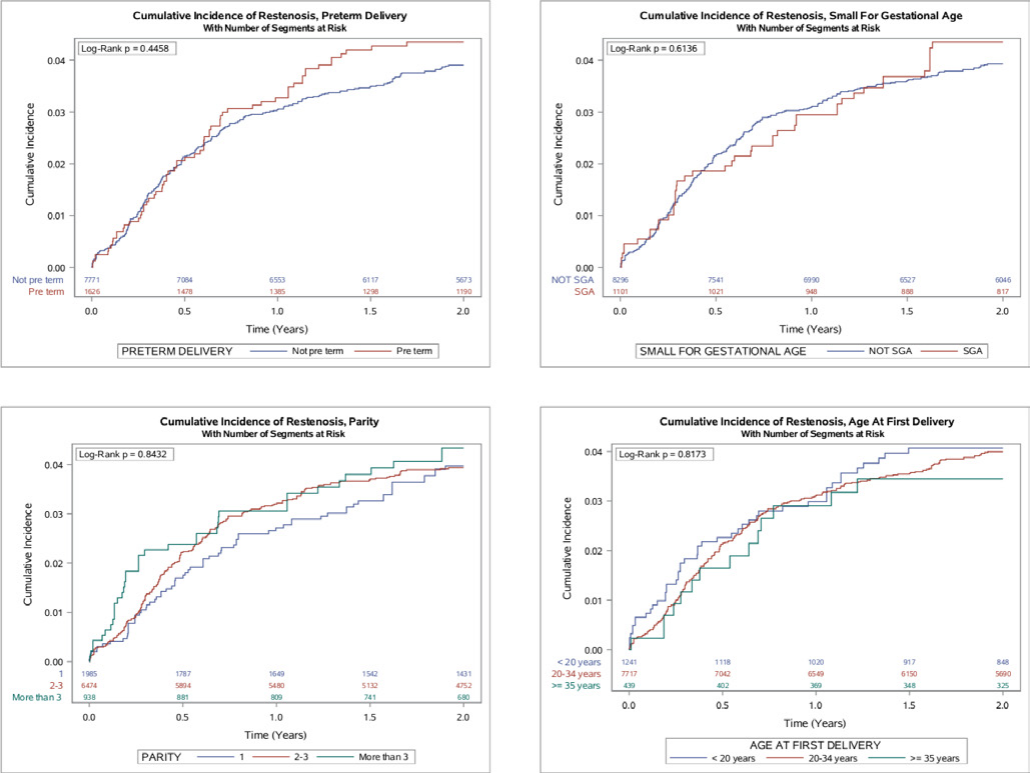
Cumulative incidence of restenosis by aspects of pregnancy history. Figure shows the unadjusted rate of restenosis by aspects of pregnancy history. Event rates were estimated using the Kaplan-Meier method and comparisons made using the log-rank test. SGA, small for gestational age.

Figure 2 shows the cumulative incidence of clinical restenosis by pregnancy history. A history of PTD was not associated with an increased unadjusted rate of clinical restenosis, nor were any of the other studied pregnancy history exposures.

Table 2 presents the results from the per-segment analysis on clinical restenosis following first PCI by pregnancy history. In total, 343 (3.7 %) events occurred following first PCI during a follow-up time of 15 981 segment-years. There was no strong evidence of an association between a history of PTD, a history of ever SGA, parity at time of PCI or age at first birth and clinical restenosis following first PCI, though point estimates for late PTD, SGA and greater parity were all greater than one.

**Table 2:** Restenosis following PCI by aspects of pregnancy history in per segment analyses

	Model I		Model II		Model III	
	HR (95% CI)	P value	HR (95% CI)	P value	HR (95% CI)	P value
Preterm delivery (PTD) (events/segment years)						
No PTD (278/13 204)	1 (reference)		1 (reference)		1 (reference)	
Ever PTD (65/2.776)	1.08 (0.77 to 1.53)	0.65	1.17 (0.83 to 1.65)	0.37	1.09 (0.77 to 1.55)	0.62
Late PTD (50/1933)	1.21 (0.82 to 1.78)	0.35	1.28 (0.87 to 1.88)	0.21	1.19 (0.81 to 1.76)	0.37
Very PTD (15/843)	0.81 (0.44 to 1.50)	0.50	0.91 (0.48 to 1.70)	0.76	0.85 (0.45 to 1.59)	0.60
Small for gestational age (SGA) (events / segment years)						
No SGA (299/14 073)	1 (reference)		1 (reference)		1 (reference)	
Ever SGA (44/1907)	1.09 (0.72 to 1.65)	0.69	1.10 (0.72 to 1.66)	0.69	1.13 (0.75 to 1.72)	0.56
Parity at time of PCI (events / segment years)						
Parity 1 (70/3332)	1 (reference)		1 (reference)		1 (reference)	
Parity 2–3 (235/11 029)	1.00 (0.71 to 1.42)	0.99	1.08 (0.76 to 1.53)	0.69	1.13 (0.79 to 1.61)	0.51
Parity >4 (38/1618)	1.09 (0.66 to 1.80)	0.73	1.22 (0.73 to 2.02)	0.45	1.27 (0.77 to 2.11)	0.35
Age at first delivery (years) (events/segment years)						
Age <20 (46/2041)	1 (reference)		1 (reference)		1 (reference)	
Age 20–34 (283/13 187)	1.00 (0.67 to 1.49)	0.99	0.90 (0.60 to 1.35)	0.63	0.90 (0.60 to 1.37)	0.63
Age >35 (14/751)	0.87 (0.43 to 1.75)	0.69	0.74 (0.36 to 1.53)	0.42	0.73 (0.35 to 1.52)	0.40

Model I: age at index PCI.

Model II: additionally accounted for indication of PCI (STEMI, NSTEMI, unstable CAD, stable CAD, other); year of procedure (2006–2009, 2010–2013, 2014–2017); treated vessel (RCA, left main, LAD, LCX, other); class of stenosis (A, B1, B2 or C); type of device(s) (BMS only, (BMS, predilation with balloon), DES only (DES, predilation with balloon),(Balloon only, drug coated) or (Balloon only, not drug coated)); length of stent; stent diameter >3 mm.

Model III: additionally accounted for diabetes; hypertension; dyslipidaemia; smoking; previous MI.

Results from multiple imputation analysis.

BMS, bare metal stent; CAD, coronary artery disease; DES, drug-eluting stent; LAD, left anterior descending coronary artery; LCX, left circumflex coronary artery; LMS, left main stem; MI, myocardial infarction; PCI, percutaneous coronary intervention; PTD, preterm delivery; RCA, right coronary artery; SGA, small for gestational age.

### TLR by pregnancy history

**Figure 3:**
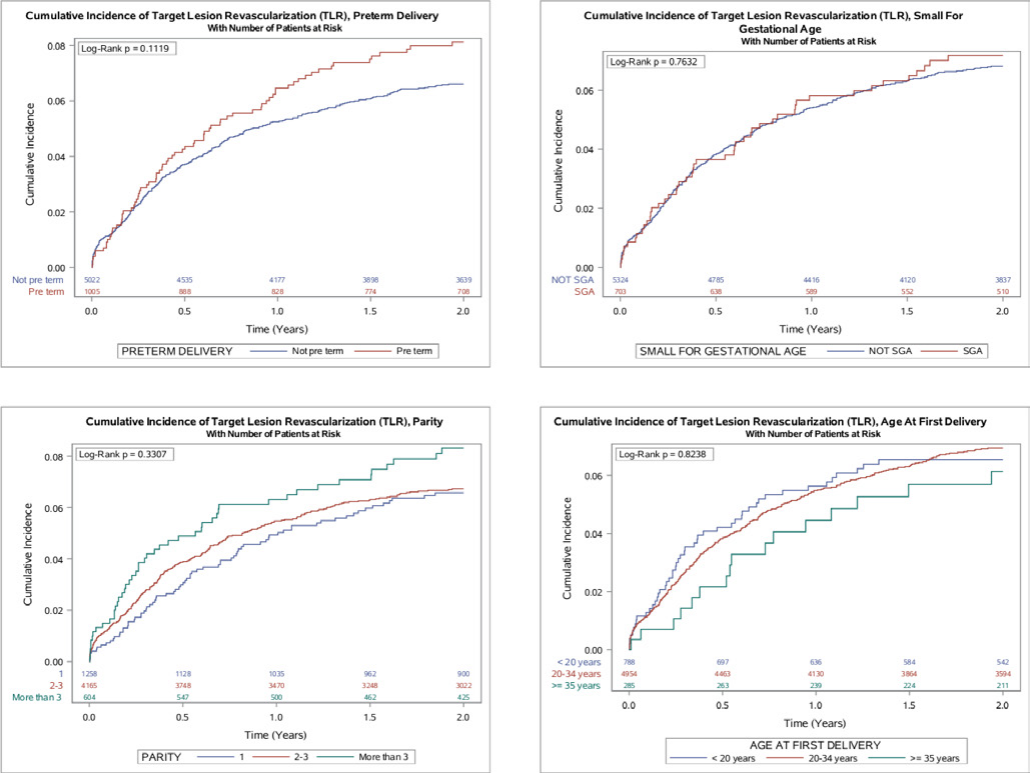
Cumulative incidence of target lesion revascularisation by aspects of pregnancy history. Figure shows the unadjusted rate of target lesion revascularisation by aspects of pregnancy history. Event rates were estimated using the Kaplan-Meier method and comparisons made using the log-rank test. SGA, small for gestational age.

Figure 3 shows the cumulative incidence of TLR by pregnancy history. None of the pregnancy history variables studied were associated with an increased unadjusted rate of TLR.

Online supplemental table 2 shows the results from the per-patient analysis on TLR by pregnancy history. In total, 383 (6.4 %) events occurred following first PCI during a follow-up time of 10 103 person-years. There was no strong evidence of associations between the studied pregnancy history variables and TLR following first PCI. Here too, point estimates for late PTD, SGA and greater parity were above one but CIs were wide.

## Discussion

In this prospective cohort study, we show that neither a history of PTD, nor several other aspects of pregnancy history, are strongly associated with clinical restenosis following PCI in parous women <65 years.

Restenosis is an adverse outcome of PCI characterised by an inflammatory response to vessel wall damage.^15^ Increased inflammatory markers, such as C reactive protein, are seen in patients who develop restenosis after PCI.^16^ The pregnancy history aspects examined in this study are associated with the development of future CAD, possibly due to inflammation and/or endothelial dysfunction.

PTD is a risk factor for future maternal CAD.^4^ As stated above, the association has been partly attributed to that PTD and the development of future CAD share common pathways.^17–19^ Up to a quarter of the association between PTD and future maternal CAD has been shown to be explained by placental disorders such as pre-eclampsia, another female-specific risk factor for future maternal CAD.^20^

Women with a history of SGA are also at an increased risk for future cardiovascular disease (CVD).^21^ Even though the pathophysiological background to this association is overall inadequately understood, it has been suggested that a delivery complicated by SGA is associated with endothelial dysfunction and thus future development of CVD. Endothelial dysfunction, and in turn placental dysfunction, is recognised as an underlying pathway for development of future maternal CVD in other pregnancy complications such as pre-eclampsia.^22–24^

Parity is associated with a non-linear increased risk of maternal CVD,^3 25^ and the increased risk in women with parity >4 could be explained by an association with subclinical atherosclerosis in these women.^26^ Pregnancy in itself can be seen as an atherogenic state with dyslipidaemia, insulin resistance and weight gain, and it has been hypothesised that a prolonged exposure time to this state is what leads to atherosclerosis and increased risk of CVD in these women.^26^ Alternatively, raising a larger family may increase CVD risk via adverse effects on diet, sleep, physical activity and other behaviours, as well as weight gain.

Studies have shown a possible inverse association between age at first delivery and future CVD.^2^ Although socioeconomic factors are highlighted as the most likely explanation, it has also been suggested that the physiological changes a pregnancy entails could affect an adolescent body differently compared with an adult body. It could also be explained by the fact that some studies show an association between low maternal age and adverse pregnancy outcomes that are known predictors of future CVD (eg, PTD and delivering an SGA infant).^27 28^

We have previously reported that PTD warrants consideration as a risk factor in the secondary prevention setting post-coronary artery stenting.^5^ Though we report no association between the exposures in this study and clinical restenosis, studies like this could still be clinically relevant. As restenosis is often associated with angina or acute coronary syndrome and patients with restenosis often undergo TLR,^15^ studies that contribute to a better understanding of groups with a higher risk of adverse outcomes post-PCI can in turn contribute to a better patient outcome. As we have previously mentioned, further studies are needed to investigate how existing strategies for secondary prevention in the post-PCI stetting can reduce the risk of adverse outcomes postcoronary artery stenting in women with a history of PTD.

### Strengths and limitations

The main strength of this study is the comprehensive and national study sample based on data from richly-completed well-known registers.^11 13^ The extensive nature of both the Medical Birth Register and SCAAR allowed us to include pregnancy data collected over decades and to adjust for several known predictors of restenosis, both procedure related and patient related. Another strength is our use of multiple imputation to account for missing data. However, this study also had some limitations. While our sample covers over a decade of national data, incident restenosis events are relatively rare, and our results do not exclude small to moderate associations for all exposures. Furthermore, we only included women age <65 years. As previously described, age is a strong predictor of worse outcome after PCI^29^ and older women have much less complete delivery history available in the Medical Birth Registry, which started in 1973. Additionally, we excluded women with PCI before 2006 as not all procedure-related variables were routinely collected before 2006. Our results are dependent on a consistency in PCI-related treatment of the women studied (eg, drug treatment before, during and after PCI). We believe an inconsistency to be unlikely given that pregnancy history is not considered in any relevant guidelines of acute cardiac care and was therefore not likely considered in the care of the women included in this study. Furthermore, we observed no major difference in the restenosis estimates by pregnancy history after adjusting for several patient-related and procedure-related predictors. The generalisation of the results could possibly be affected by the ethnic homogeneity of the study sample. Lastly, it should be mentioned that pregnancy dating using ultrasound was not widely used in Sweden until the 1970s, and at the beginning of the Medical Birth Register not all pregnancies were dated using ultrasound.

## Conclusion

In conclusion, neither a history of PTD nor the other aspects of pregnancy history studied were associated with clinical restenosis following PCI. However, larger studies are needed to obtain more precise estimates.

## Data Availability

No data are available.
