# Supplementary material for: Coronary artery restenosis and target lesion revascularisation in women by pregnancy history
Source: Open Heart. 2023 Mar 17;10(1):e002130. doi: 10.1136/openhrt-2022-002130 (PMC10030621; doi:10.1136/openhrt-2022-002130)
Supplement: Supplementary data [file openhrt-2022-002130supp001.pdf]

ONLINE SUPPLEMENT

Coronary artery restenosis and target lesion revascularization in women by pregnancy history

Pehrson et al.

Supplemental tables

Supplemental Table 1. Descriptive characteristics per segment at time of index PCI by aspects of pregnancy history (n=9,397)

Supplemental Table 2. Target lesion revascularization following PCI by aspects of pregnancy history in per patient analyses

Supplemental Table 1. Descriptive characteristics per segment at time of index PCI by aspects of pregnancy history (n=9,397)

	**Preterm delivery**		**Small for gestational age**		**Parity at time of PCI**			**Age at first delivery (years)**		
**Variables n, (%) unless stated**	No PTD (n=7,771)	Ever PTD (n=1,626)	No SGA (n=8,296)	Ever SGA (n=1,101)	Parity 1 (n=1,985)	Parity 2-3 (n=6,474)	Parity >4 (n=983)	Age <20 (n=1,241)	Age 20-34 (n=7,717)	Age ≥35 (n=439)
Class of stenosis										
Type A	936 (12.0)	202 (12.4)	994 (12.0)	144 (13.1)	191 (9.6)	832 (12.9)	115 (12.3)	159 (12.8)	927 (12.0)	52 (11.9)
Type B1	3,049 (39.2)	581 (35.7)	3,235 (39.0)	395 (35.9)	757 (38.1)	2,497 (38.6)	376 (40.1)	462 (37.2)	2,984 (38.7)	184 (41.9)
Type B2	2,490 (32.0)	542 (33.3)	2,679 (32.3)	353 (32.1)	674 (34.0)	2,075 (32.1)	283 (30.2)	380 (30.6)	2,531 (32.8)	121 (27.6)
Type C	1,296 (16.7)	301 (18.5)	1,388 (16.7)	209 (19.0)	363 (18.3)	1,070 (16.5)	164 (17.5)	240 (19.3)	1,275 (16.5)	82 (18.7)
Treated vessel										
RCA	2,522 (32.5)	573 (35.2)	2,680 (32.3)	415 (37.7)	662 (33.4)	2,125 (32.8)	308 (32.8)	470 (37.9)	2,512 (32.6)	113 (25.7)
LMS	85 (1.1)	20 (1.2)	94 (1.1)	11 (1.0)	22 (1.1)	73 (1.1)	10 (1.1)	13 (1.1)	87 (1.1)	5 (1.1)
LAD	3,639 (46.8)	701 (43.1)	3,900 (47.0)	440 (40.0)	914 (46.1)	2,996 (46.3)	430 (45.8)	519 (41.8)	3,593 (46.6)	228 (51.9)
LCX	1,313 (16.9)	279 (17.2)	1,386 (16.7)	206 (18.7)	333 (16.8)	1,095 (16.9)	164 (17.5)	209 (16.8)	1,303 (16.9)	80 (18.2)
Other	212 (2.7)	53 (3.3)	236 (2.8)	29 (2.6)	54 (2.7)	185 (2.9)	26 (2.8)	30 (2.4)	222 (2.9)	13 (3.0)
Type of device(s) used										
BMS only	619 (8.0)	148 (9.1)	675 (8.1)	92 (8.4)	159 (8.0)	543 (8.4)	65 (6.9)	83 (6.7)	644 (8.4)	40 (9.1)
BMS, predilation with balloon	1,377 (17.7)	231 (14.2)	1,407 (17.0)	201 (18.3)	353 (17.8)	1,101 (17.0)	154 (16.4)	191 (15.4)	1,338 (17.3)	79 (18.0)
DES only	1,255 (16.2)	244 (15.0)	1,313 (15.8)	186 (16.9)	277 (14.0)	1,061 (16.4)	161 (17.2)	214 (17.2)	1,201 (15.6)	84 (19.1)
DES, predilation with balloon	3,912 (50.3)	884 (54.4)	4,265 (51.4)	531 (48.2)	1,029 (51.8)	3,288 (50.8)	479 (51.2)	667 (53.8)	3,923 (50.8)	206 (46.9)
Balloon only, drug coated	130 (1.7)	33 (2.0)	139 (1.7)	24 (2.2)	37 (1.9)	114 (1.8)	12 (1.3)	17 (1.4)	142 (1.8)	4 (0.9)
Balloon only, not drug coated	478 (6.2)	86 (5.3)	497 (6.0)	67 (6.1)	130 (6.6)	367 (5.7)	67 (7.1)	69 (5.6)	469 (6.1)	26 (5.9)
Length of stent(s) if used (SD)	19.2 (7.4)	19.5 (7.8)	19.3 (7.5)	19.2 (7.3)	19.3 (7.5)	19.3 (7.4)	19.3 (7.8)	19.6 (7.9)	19.3 (7.4)	18.6 (7.5)
Stent diameter >3 mm if stent used	1,913 (26.7)	410 (27.2)	2,036 (26.6)	287 (28.4)	467 (25.7)	1,615 (27.0)	241 (28.1)	324 (28.1)	1,894 (26.7)	105 (25.7)

BMS: bare metal stent; DES: drug eluting stent; LAD: left anterior descending coronary artery; LCX: left circumflex coronary artery; LMS: left main stem; PCI: percutaneous coronary intervention; PTD: preterm delivery; RCA: right coronary artery; SGA: small for gestational age; SD: standard deviation.
Information presented per segment. No missing.

Supplemental Table 2. Target lesion revascularization following PCI by aspects of pregnancy history in per patient analyses

	**Model I**		**Model II**		**Model III**	
	HR (95% CI)	*p*	HR (95% CI)	*p*	HR (95% CI)	*p*
**Preterm delivery (PTD)** (events / person years)						
No PTD (308 / 8,436)	1 (reference)		1 (reference)		1 (reference)	
Ever PTD (75 / 1,666)	1.20 (0.93-1.55)	0.16	1.22 (0.95-1.58)	0.12	1.18 (0.91-1.52)	0.21
Late PTD (54 / 1,130)	1.28 (0.96-1.71)	0.09	1.28 (0.96-1.71)	0.10	1.24 (0.92-1.66)	0.15
Very PTD (21 / 536)	1.04 (0.67-1.61)	0.88	1.11 (0.71-1.72)	0.66	1.05 (0.67-1.64)	0.83
**Small for gestational age (SGA)** (events / person years)						
No SGA (336 / 8,914)	1 (reference)		1 (reference)		1 (reference)	
Ever SGA (47 / 1,189)	1.05 (0.77-1.42)	0.78	1.05 (0.77-1.42)	0.77	1.05 (0.77-1.42)	0.77
**Parity at time of PCI** (events / person years)						
Parity 1 (75 / 2,093)	1 (reference)		1 (reference)		1 (reference)	
Parity 2 – 3 (261 / 7,002)	1.03 (0.80-1.33)	0.82	1.08 (0.83-1.39)	0.57	1.11 (0.86-1.44)	0.43
Parity $\geq$ 4 (47 / 1,007)	1.27 (0.88-1.82)	0.21	1.39 (0.96-2.01)	0.08	1.43 (0.99-2.07)	0.06
**Age at first delivery (years)** (events / person years)						
Age < 20 (48 / 1,283)	1 (reference)		1 (reference)		1 (reference)	
Age 20 – 34 (319 / 8,332)	1.08 (0.79-1.46)	0.64	1.00 (0.73-1.36)	0.99	1.01 (0.74-1.37)	0.97
Age $\geq$ 35 (16 / 487)	0.92 (0.52-1.62)	0.78	0.81 (0.46-1.43)	0.47	0.82 (0.46-1.44)	0.48

BMS: bare metal stent; DES: drug eluting stent; LAD: left anterior descending coronary artery; LCX: left circumflex coronary artery; LMS: left main stem; MI: myocardial infarction; PCI: percutaneous coronary intervention; PTD: preterm delivery; SGA: small for gestational age
Model I: age at index PCI
Model II: additionally accounted for indication of PCI (STEMI, NSTEMI, unstable CAD, stable CAD, other); year of procedure (2006 – 2009, 2010 – 2013, 2014 – 2017); treated vessel (RCA, left main, LAD, LCX, other); class of stenosis (A, B1, B2, or C); type of device(s) (BMS only, [BMS, predilation with balloon], DES only, [DES, predilation with balloon], [Balloon only, drug coated], or [Balloon only, not drug coated]); length of stent; stent diameter >3mm
Model III: additionally accounted for diabetes; hypertension; dyslipidaemia; smoking; previous MI
Results from multiple imputation analysis
